# Integration of Immune Cell-Target Cell Conjugate Dynamics Changes the Time Scale of Immune Control of Cancer

**DOI:** 10.1007/s11538-024-01400-2

**Published:** 2025-01-03

**Authors:** Qianci Yang, Arne Traulsen, Philipp M. Altrock

**Affiliations:** Department of Theoretical Biology, Max Planck Institute for Evolutionary Biology, August-Thienemann-Strasse 2, 24306 Ploen, Germany

**Keywords:** Mathematical modeling, Conjugate, Effector T cells, Cancer immune dynamics

## Abstract

The human immune system can recognize, attack, and eliminate cancer cells, but cancers can escape this immune surveillance. Variants of ecological predator–prey models can capture the dynamics of such cancer control mechanisms by adaptive immune system cells. These dynamical systems describe, e.g., tumor cell-effector T cell conjugation, immune cell activation, cancer cell killing, and T cell exhaustion. Target (tumor) cell-T cell conjugation is integral to the adaptive immune system’s cancer control and immunotherapy. However, whether conjugate dynamics should be explicitly included in mathematical models of cancer-immune interactions is incompletely understood. Here, we analyze the dynamics of a cancer-effector T cell system and focus on the impact of explicitly modeling the conjugate compartment to investigate the role of cellular conjugate dynamics. We formulate a deterministic modeling framework to compare possible equilibria and their stability, such as tumor extinction, tumor-immune coexistence (tumor control), or tumor escape. We also formulate the stochastic analog of this system to analyze the impact of demographic fluctuations that arise when cell populations are small. We find that explicit consideration of a conjugate compartment can (i) change long-term steady-state, (ii) critically change the time to reach an equilibrium, (iii) alter the probability of tumor escape, and (iv) lead to very different extinction time distributions. Thus, we demonstrate the importance of the conjugate compartment in defining tumor-effector T cell interactions. Accounting for transitionary compartments of cellular interactions may better capture the dynamics of tumor control and progression.

## Introduction

Cancer has been one of the major health and clinical challenges worldwide and is a significant contributor to mortality (Bray et al. 2024). The human body can command various defences against cancer growth. Some are cell-intrinsic, such as the apoptotic machinery, which triggers the death of damaged or mutated cells. Tissues can also restrict the growth of developing cancer cell populations, for example, through hierarchical organization into stem, progenitor, and differentiated cell compartments (Nowak et al. 2003; Wodarz and Komarova 2005; Werner et al. 2013; Hindersin et al. 2016), or by spatial constraints on the spread of mutants (Komarova et al. 2014; Wodarz and Komarova 2020). Apart from these more ’passive’ cancer suppression mechanisms, the human immune system can actively recognize, attack, and eliminate cancer cells (Munhoz and Postow 2016). During tumorigenesis, cancers may escape from this immune surveillance, but modern therapies can reactivate anti-cancer immune activity (Waldman et al. 2020). The dynamics of these cancer control mechanisms, e.g., exerted by the adaptive immune system (Gonzalez et al. 2018), can be captured by predator–prey models and related dynamical systems from ecology (Kareva et al. 2021; Hamilton et al. 2022).

Effector T cells are essential to the human adaptive immune system’s ability to tag and kill aberrant cells (Raskov et al. 2021). These aberrant tumor cells often generate neoantigens due to tumor-specific molecular alterations, such as mutations, which are recognized as non-self. A neoantigen is a protein appearing on the membrane of immunogenic tumor cells and presents a target to trigger an immune response in the host (Xie et al. 2023). Effector T cells can be activated by recognizing neoantigens and killing target cells (Weinberg and Weinberg 2006). This recognition is achieved by the antigen-presenting cells, which display the antigen to provoke the effector T cell’s activation (Vinay et al. 2015), yet also lead to exhaustion in the long run (Raskov et al. 2021). The activated effector T cell proliferates and uses the T cell receptor on its membrane to recognize and bind to the antigen presented on the surfaces of the targeted tumor cells (Wang et al. 2019). The so-formed conjugation enables the effector T cell to deliver a potentially lethal hit to the target tumor cell. Thus, T cell-target cell conjugation is integral to the adaptive immune system’s cancer control or immunotherapy dynamics, as conjugation dynamics may lead to a variation of cytotoxic efficiency (Weigelin et al. 2021). Whether conjugate dynamics should be explicitly captured in mathematical models of cancer-immune interactions is incompletely understood.

Mathematical modeling of biological systems provides powerful tools for formalizing and testing theories (Wodarz and Komarova 2014; Altrock et al. 2015), explaining empirical observations combining mechanisms that were thought to be unrelated (Otto and Day 2007; Hart et al. 2014; Kimmel et al. 1947; Gerlee et al. 2022), and explicitly for understanding and exploring the interaction of the immune system and cancer (Robertson-Tessi et al. 2012; Wilkie 2013; Swanson et al. 2022; Butner et al. 2022). Kuznetsov et al. introduced a mathematical model of the cytotoxic T lymphocyte response to the growth of an immunogenic tumor (Kuznetsov et al. 1994). The model involves effector cells, tumor cells, and their formed conjugates that consist of a pair of cells (one tumor or target cell and one effector cell). This conjugate population was then assumed to equilibrate very fast compared to the overall dynamics, subtracting their active contribution to the observed dynamics of tumor burden. Mathematical models that explicitly consider the influence of conjugate dynamics on tumors and immune interactions can provide another perspective for analyzing such systems. Here, we ask under which circumstances the conjugate compartment can assumed to be in such a quasi-steady state in a small tumor. We compare a mathematical model consisting of effector T cells and target tumor cells (two-compartment-model, without conjugate compartment) to a system composed of effector T cells, target tumor cells, and conjugates (three-compartment-model, with a conjugate compartment). We first analyze the deterministic dynamics of these two models: We apply linear stability analysis, investigate the ODE time series damping rate, and quantify the time for the system’s approach to equilibrium. Second, we consider a stochastic approach for both models to describe the dynamics of small populations that are subject to demographic fluctuations. This leads to a formulation of the tumor eradication time and allows us to assess how parameter variability influences the associated time distribution.

## Methods

### Deterministic Dynamics

**Fig. 1:**
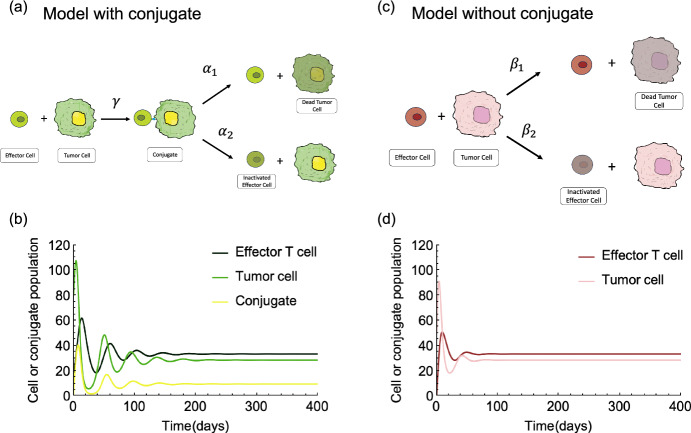
Model with conjugate and Model without conjugate. (**a**) Schematic drawing of effector T cell, target tumor cell, and conjugate system (model with conjugate). (**b**) Deterministic dynamics of the model with conjugate (parameters $s = 0.15$, $r =0.3$, $d=0.1$, $\gamma =0.01$, $\alpha _1 = 0.9$, and $\alpha _2=0.1$). (**c**) Schematic drawing of the effector T cell and the target tumor cell system (model without conjugate). (**d**) Deterministic dynamics of the model without conjugate (parameters $s = 0.15$, $r =0.3$, $d=0.1$, $\beta _1 = 0.009$, $\beta _2=0.001$)

Models describing the dynamics of tumor cells and effector cells mostly focus on the direct interaction of these populations (Kuznetsov et al. 1994). In contrast, we compare a model with a conjugate compartment to a model without that compartment to emphasize the impact of conjugate on the system. We consider the scheme in Fig. 1a to model the interaction of effector T cells and target tumor cells and effector T cell binding with the target tumor cell by matching T cell receptor and proteins on the tumor surface. Subsequently, conjugation either leads to the death of the tumor cell or the exhaustion of the effector T cell.

The model consists of the variables *E* (effector T cell), *T* (target tumor cell), *C* (conjugate), $E^{\dagger }$ (exhausted effector T cell) and $T^{\dagger }$ (dead tumor cell).

The parameters $\gamma$, $\alpha _1$ and $\alpha _2$ are non-negative constants: $\gamma$ is the rate of effector T cell and tumor cell conjugate formation, $\alpha _1$ is the rate at which conjugate leads to tumor cell lysis, and $\alpha _2$ represents the effector T cell inactivation rate. Failure to lyse tumor cells results in persistent antigen stimulation. Under such conditions, effector cells gradually become functionally exhausted (Eftimie et al. 2011; Chi et al. 2024). The population dynamics of effector T cell and tumor cell are described by

1a $$\begin{aligned} \frac{\textrm{d}E}{\textrm{d}t}&=sT-\gamma ET+\alpha _1C-dE \end{aligned}$$

1b $$\begin{aligned} \frac{\textrm{d}T}{\textrm{d}t}&= rT - \gamma ET+\alpha _2C \end{aligned}$$

1c $$\begin{aligned} \frac{\textrm{d}C}{\textrm{d}t}&= \gamma ET-\alpha _1C-\alpha _2C \end{aligned}$$

The reproduction term of effector T cells is *sT*. Iezzi et al. (1998) observed that naive T cells are stimulated to proliferate under the signaling information conveyed by antigen-presenting cells. This suggests that the influx of mature effector T cells depends on the target tumor cell population *T*, the constant number of naive cells *N* and a constant differentiation rate $\sigma$. We use a positive coefficient *s* to represent the product of *N* and $\sigma$, multiplying the population of target cells to express the influx of mature effector T cells. Effector T cells are removed at rate $d > 0$, due to natural death or migration out of the tumor location. The term *rT* describes the exponential growth of the target tumor cell. The system consists of the effector T cell, target tumor, and conjugates, which we call "model with conjugate" for short, see Fig. 1a.

Forming a conjugate before target tumor cell lysis and effector T cell exhaustion corresponds to a handling time problem. To investigate whether the handling time affects the dynamics of the system and, if so, to what extent, we modify the model by applying a quasi-steady state approximation, assuming that the dynamics of the conjugate occur on a much faster time scale compared to those of effector T cell and target tumor cell. This approximation implies setting the differential equation for the conjugate to zero, leading to an expression for the conjugates as a function of *E* and *T*

2 $$\begin{aligned} C = \frac{\gamma }{\alpha _1+\alpha _2}ET \end{aligned}$$

Substituting Eq. (2) into Eq. (1a) and Eq. (1b), we obtain a dynamical system in the limit that conjugate compartment dynamic is infinitely fast - a model without conjugate (two compartments),

3a $$\begin{aligned} \frac{\textrm{d}E}{\textrm{d}t}&=sT-\gamma \frac{\alpha _2}{\alpha _1+\alpha _2}ET-dE \end{aligned}$$

3b $$\begin{aligned} \frac{\textrm{d}T}{\textrm{d}t}&= rT - \gamma \frac{\alpha _1}{\alpha _1+\alpha _2}ET \end{aligned}$$

With $\beta _2 = \gamma \frac{\alpha _2}{\alpha _1+\alpha _2}$ and $\beta _1 = \gamma \frac{\alpha _1}{\alpha _1+\alpha _2}$, we have a deterministic model for the system without conjugate (see Fig. 1c),

4a $$\begin{aligned} \frac{\textrm{d}E}{\textrm{d}t}&= sT-\beta _2ET-dE \end{aligned}$$

4b $$\begin{aligned} \frac{\textrm{d}T}{\textrm{d}t}&= rT -\beta _1ET \end{aligned}$$

### Stochastic Dynamics

The deterministic version of the model describes the dynamics of large cell populations. However, it does not accurately represent the dynamics of small cell populations in which stochastic effects can dominate. A stochastic model can better capture the dynamics of small cell populations, as every reaction is explicitly accounted for. We implemented the Gillespie algorithm to simulate stochastic equation system trajectories (Gillespie 1977). The Gillespie algorithm is a variant of a dynamic Monte Carlo method and generates a realization of the Master equation, which is the first order in time interval of Markov processes in continuous time. Each state change is the reaction, and the corresponding propensity functions are the likelihood of the reaction happening in a short time interval. The chemical reactions with corresponding propensity functions for models with conjugate and without conjugate are listed in Table 1. The variables *E*, *T*, and *C* are used for the same populations as in the deterministic model. $E^{\dagger }$ and $T^{\dagger }$ represent inactivation effector cells and dead tumor cells. In the left part of Table 1, parameters *d*, *s*, and *r* represent the reaction rate of one effector cell’s death, the reaction rate of one effector birth when tumor cells present, and the reaction rate of one tumor cell birth. The parameter $\gamma$ is the reaction rate of one effector cell and one tumor cell binding. The reaction rates that one conjugate leads to either the tumor cell being killed or the effector cell inactivation are $\alpha _1$ and $\alpha _2$. The propensity functions, also called transition probabilities, are based on the number of cells present in the system. We assume the volume of the system *v* is one. In Table 1 right table, parameters *d*, *s* and *r* have the same meaning as those in the left part of Table 1. Here, there are two possible events when one effector cell meets one tumor cell, represented by $\beta _1$ and $\beta _2$. While $\beta _1$ describes the reaction rate of effector cell-tumor cell coming together resulting in tumor cell killing, the parameter $\beta _2$ is the reaction rate of effector cell and tumor cell coming together resulting in tumor cell survival and effector T cell exhaustion.

These reactions describe stochastic processes that allow the derivation of mean-field dynamical systems. To connect with the deterministic models in the form of a system size expansion, we apply the master equation of the stochastic models and arrive at the Fokker-Planck equation. The details are in part A of the Appendix.

**Table 1:** Table of chemical reactions and corresponding propensity functions for model with conjugate and model without conjugate

Reaction (model with conjugate)	Propensity Function (model with conjugate)
$E \rightarrow \emptyset$	*dE*
$T \rightarrow T+E$	*sT*
$T \rightarrow T+T$	*rT*
$E+T \rightarrow C$	$\gamma ET/v$
$C \rightarrow T+E^{\dagger }$	$\alpha _1 C$
$C \rightarrow T^{\dagger }+E$	$\alpha _2 C$

Reaction (model without conjugate)	Propensity Function (model without conjugate)
$E \rightarrow \emptyset$	*dE*
$T \rightarrow T+E$	*sT*
$T \rightarrow T+T$	*rT*
$E+T \rightarrow T^{\dagger }+E$	$\beta _1 ET/v$
$E+T \rightarrow T+E^{\dagger }$	$\beta _2 ET/v$

### Parameter Choices

The parameter units are as follows: for the binding rate $\gamma$, the unit is $day^{-1} cells^{-1}$. The units of effector cell reproduction rate *s*, tumor cell reproduction rate *r*, the effector cell death rate *d*, the tumor-killing rate $\alpha _1$, $\beta _1$, and the effector cell inactivation rate $\alpha _2$, $\beta _2$ are $day^{-1}$.

To explore the parameters space, we determine the values of parameters by varying values over certain biologically reasonable ranges. In stochastic simulation, we set the default value of *s* to be 0.05, as this highly differentiated T cell influx rate is usually greater than the naive and memory T cell reproduction rates. Macallan et al. (2019) The value of *s* varies from 0.05 to 0.25 to explore the tumor extinction probability and time distribution. For *r*, we set its range to be 0.1 to 0.3. Ribba et al. (2012) We vary the effector cell death rate *d*, from 0.02 to 0.1 to see if the extinction is affected if the effector cell death rate is increasing. Ribba et al. (2012) The binding rate is set to be low (0.02 to 0.12) to investigate the transition stage’s influence. Another fact we take into account is that the 1 : 1 effector cell and tumor cell pairing is not always the conjugate form in reality, more effector cell-binding events can happen to kill one tumor cell. Weigelin et al. (2021) Hence, we lower the binding rate to compensate for this assumption effect. We choose the tumor-killing rate $\alpha _1$ to be much greater than the effector cell inactivation rate $\alpha _2$, determining the value considering (Weigelin et al. 2021). The parameter choices in our model without conjugate follow the same strategy. In the deterministic model simulation, the parameter values are set to be greater since we aim to compare the equilibrium convergence speed by recording the time. By the parameter combination in Eq. (4b), the values of $\beta _1$ and $\beta _2$ are determined by the values of $\gamma$, $\alpha _1$ and $\alpha _2$.

We investigate the characteristic times by non-dimensionalising both deterministic models, Eqs. (1c) and (4b). It turns out that *d* is the common denominator of all rescaled parameters in the non-dimensionalised models. Details are summarised in part B in the Appendix.

## Results

### Explicitly Modeling Conjugate Compartments Can Change the Stability of Steady States

To compare cancer-immune dynamics with and without immune cell-cancer cell conjugation, we first focus on the steady states. Both systems have two steady states, either extinction or coexistence of all described cell types. Note that a condition $\alpha _1s-\alpha _2r > 0$ must be satisfied to make all steady states feasible (i.e. population sizes are not negative). We summarise the stability of steady states in Table 2. The extinction fixed points are unstable saddle points in both systems. For the model without conjugate, the coexistence steady state is stable as the eigenvalue of the Jacobian matrix always has negative real part ( proof in Appendix part C: 1). The phase portrait of the model without conjugate is in Fig. 3a. The stability of the coexistence steady state of the system with conjugate is less straightforward to analyze, as the eigenvalues of the Jacobian matrix are the roots for a cubic polynomial function. Numerical methods are used to determine stability (see Appendix part C: 2).

**Table 2:** Table of steady states in systems with and without conjugate and corresponding stability behavior

Model	Steady state	Stability
Model with conjugate	(0, 0, 0)	saddle point
	$\left( \frac{r}{\gamma }\frac{\alpha _1+\alpha _2}{\alpha _1}, \frac{dr}{\gamma }\frac{\alpha _1+\alpha _2}{\alpha _1s-\alpha _2r}, \frac{dr^2}{\gamma \alpha _1}\frac{\alpha _1+\alpha _2}{\alpha _1s-\alpha _2r} \right)$	stable / unstable
Model without conjugate	(0, 0)	saddle point
	$\left( r\frac{\alpha _1+\alpha _2}{\gamma \alpha _1}, \frac{dr}{\gamma }\frac{\alpha _1+\alpha _2}{\alpha _1s-\alpha _2r} \right)$	stable node/spiral

We express the steady states in the order (*E*, *T*, *C*) for the model with conjugate and in the order (*E*, *T*) for the model without conjugate

By comparing the coexistence steady states of two models, we find that in the model without conjugate, the tumor population is under control as the coexistence is always stable. With the conjugate, the coexistence steady state can be either stable or unstable, depending on the parameter choices. Figure 7 illustrates a situation in which the tumor cell killing rate $\alpha _1$ changes the stability of the coexistence fixed point. We vary values of effector exhaustion rate and tumor-killing rate, fixing other parameters, and investigate the stability of the co-existence (non-trivial) fixed point by looking at the eigenvalues of Jacobian matrix of the ode system. We found that when we increase the effector exhaustion rate, the feasible fixed point becomes stable regardless the value of tumor-killing rate. For a relatively small effector exhaustion rate, the fixed point is unstable when tumor-killing rate is small and becomes stable tumor control for a greater tumor-killing rate.

### Taking Into Account a Conjugate Compartment Affects Time Scales

From the previous section, the populations for effector and tumor cells have the same steady states for the models with and without a conjugate compartment, but the stability of the coexistence states can differ. In this section, we focus on the time from the initial condition to the two systems’ stable, non-trivial, steady state. We are interested in how the transitional conjugate will influence the time to converge to a stable situation where a small tumor could exist. In Fig. 1b and d, we can see the ODE trajectories oscillate for a longer time until they reach the equilibrium in the model with conjugate. As the binding of effector and tumor cells has to happen first, this process leads to a longer fluctuation period to reach a stable coexistence steady state. Two features are important in characterizing the oscillation before the convergence to the fixed point: the amplitude and the damping rate. The oscillation amplitude depends on the difference between initial conditions and the population at steady state. The damping rate depends on the largest real part of the eigenvalues of the ODE’s Jacobian matrix.

**Fig. 2:**
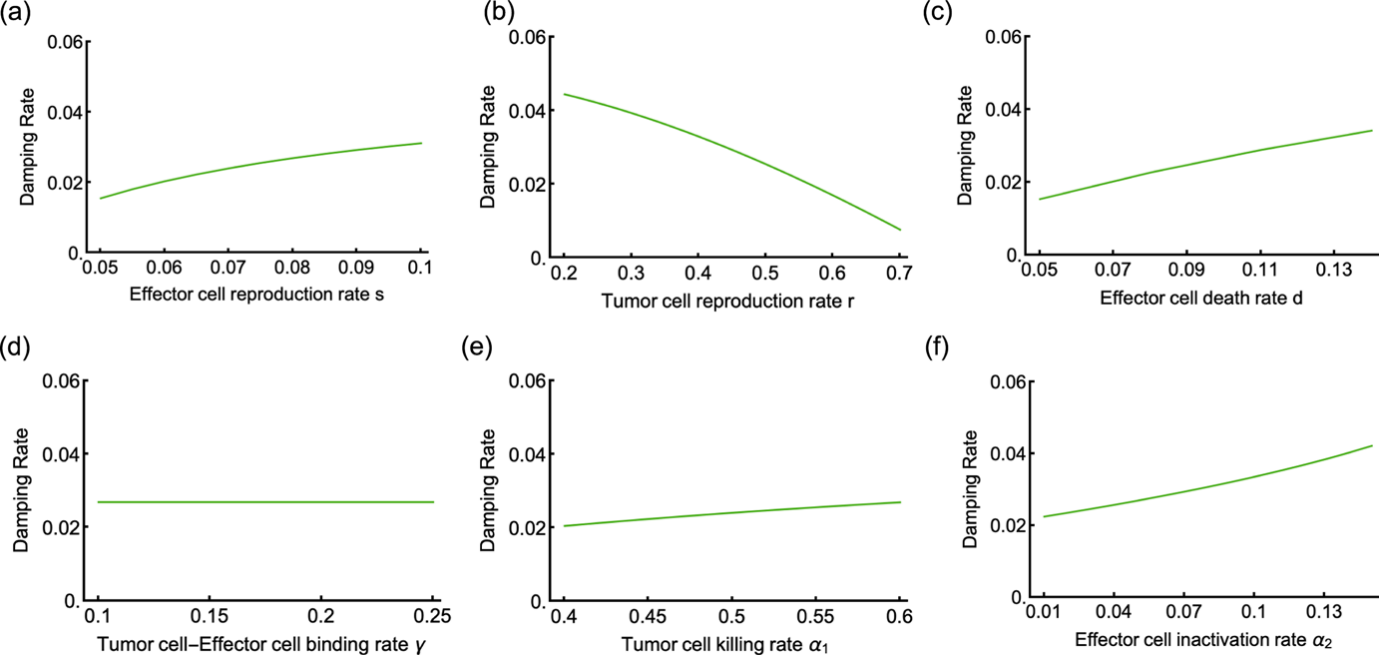
Damping rate in the model with conjugate. The damping rate of the ordinary differential equation trajectories oscillation is the largest real part of the eigenvalues of the Jacobian matrix of the ODE. Six panels show the relation between all six parameters and the absolute value of damping rates. The default six parameters are $s=0.08$, $r = 0.15$, $d = 0.1$, $\gamma =0.1$, $\alpha _1 = 0.6$, and $\alpha _2 = 0.05$. In each of the panels, we vary a single one of these parameters: In panel (**a**), we vary the effector cell reproduction rate *s*, in panel (**b**), we vary the tumor cell reproduction rate *r*, in panel (**c**), we vary the effector cell death rate *d*, in panel (**d**), we vary the effector-tumor cell binding rate $\gamma$, in panel (**e**), we vary the tumor cell killing rate $\alpha _1$, and in panel (**f**), we vary the effector cell inactivation rate $\alpha _2$. The damping rate decreases with the tumor cell reproduction rate *r*, independent of the binding rate $\gamma$, and increases with all other parameters

The eigenvalues of the Jacobian matrix for the model with a conjugate ODE are too complex to elucidate the relation between them and parameters analytically. But a numerical method reveals which parameter influences the damping rate of the ODE trajectory oscillation. Figure 2 illustrates the relation of the absolute value of damping rate and the parameters: the reproduction rate *s* of the effector cell and the reproduction rate *r* of the tumor cell, the effector cell death rate *d*, the binding rate $\gamma$, the tumor-killing rate $\alpha _1$, and the effector cell inactivation rate $\alpha _2$. Effector cell and tumor cell binding rate $\gamma$ is the only parameter that does not affect the damping rate. An increase in the tumor cell reproduction rate *r* leads to a decrease in the damping rate. This means greater tumor cell reproduction rate results in slower convergence to a co-existence equilibrium. For other parameters, the greater the parameter value, the larger the absolute value of the damping rate - leading to a in faster convergence to a co-existence equilibrium.

To visualize the time from the initial condition to the stable coexistence steady state in both systems, we run ordinary differential equations numerical solution under pairs of parameters and record the time until ordinary differential equations trajectories have reached the stable non-trivial steady states. As a criterium to end the numerical solution, we define

5 $$\begin{aligned} (E-E^*)^2+(T-T^*)^2 \le \varepsilon . \end{aligned}$$

Here, $E^*$ and $T^*$ are the population quantities for effector cell and tumor cell populations at the coexistence steady state.

**Fig. 3:**
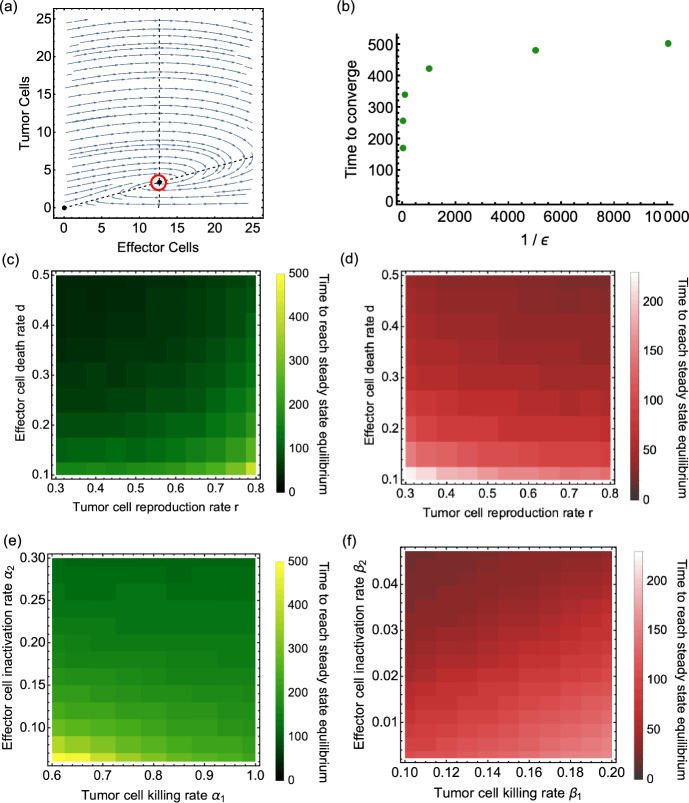
Time to reach the coexistence steady states in both systems. Panel (**a**) is the 2-dimensional phase portrait of the system without conjugate and a sketch of the threshold $\varepsilon$ (red circle). When the simulated ODE trajectories approach the threshold, the time it takes from the start to reach the red circle is recorded as the time to converge. Panel (**b**) shows the time to converge as a function of $1/\varepsilon$. We take the time points from the initial condition ($E=100$, $T=500$) to reach the threshold around non-trivial coexistence steady state in the model with conjugate. Parameters: $s = 0.6$, $r = 0.8$, $d =0.2$, $\gamma = 0.08$, $\alpha _1 = 0.5$, $\alpha _2 = 0.1$. We choose $\varepsilon = 0.01$ in the following. Panel (**c**) and (**e**) depict the time from the initial condition to reach the threshold around the coexistence steady state in the model with conjugate varying parameters. For (**c**), $s = 0.6$, $\gamma = 0.08$, $\alpha _1 = 0.8$, $\alpha _2 = 0.1$. For (**e**), $s = 0.6$, $r = 0.8$, $d = 0.2$, $\gamma = 0.08$. Panel (**d**) is the time to the non-trivial steady state when varying target tumor reproduction rate *r* and effector T cell death rate *d* in the model without conjugate, setting $s = 0.6$, $\beta _1 = 0.071$, $\beta _2 = 0.009$. Panel (f) is the convergence time in different combinations of tumor cell killing rate $\beta _1$ and effector cell inactivation rate $\beta _2$ in the model without conjugate, setting $s = 0.6$, $r = 0.8$, $d = 0.2$. The initial conditions for panels (**c**), (**d**), (**e**), and (**f**) are $E= 100$, $T= 500$, and $C= 0$ for panels (**c**) and (**e**)

We sketch the metric in the phase portrait of the model without conjugate compartment (Fig. 3a). When the simulated ODE trajectories come close to the fixed point, the convergence time is the time in which the sum of the squared difference becomes smaller than $\varepsilon$. Figure 3b shows the time it takes from the initial condition to the coexistence steady state, choosing different values of $1/\varepsilon$. The convergence time increases with $1/\varepsilon$ roughly like a logarithm. This means the time does not increase by the same amount as the reciprocal of $\varepsilon$ when $\varepsilon$ is sufficiently small. We choose 0.01 for $\varepsilon$. We set the coexistence steady state to be the population at time equals 800 days. Pairs of parameters in the model with conjugate are varied to see their influence on the converge time. Figure 3c presents the convergence time (in color scale) varying the tumor cell reproduction rate *r* and the effector cell death rate *d*. The convergence time increases with the tumor reproduction rate; this also validates the result that the damping rate is negatively correlated with the tumor reproduction rate, thus leading to slower convergence. In contrast, the growth of the death rate of effector cell *d* results in the decrease of converge time. The reduction of convergence time happens for increasing tumor killing rate $\alpha _1$ and the effector cell inactivation rate $\alpha _2$, and they are displayed in Fig. 3e. Compared to the model with conjugate, the tumor cell reproduction rate *r* plays a different role in convergence time in the model without conjugate. With the decline of *r*, it takes longer to reach the fixed point. Effector cell death rate *d* plays a consistent role in both systems, as Fig. 3d shows. The tumor cell killing rate $\beta _1$ positively correlates with time to converge, while the effector inactivation rate $\beta _2$ does not. Figure 3 panel (f) depicts the converge time change with variation of $\beta _1$ and $\beta _2$.

### Conjugate Changes Tumor Extinction Time and Tumor Can Avoid Extinction Without Conjugate

The tumor and effector cells can coexist in deterministic models. At very low levels, this may lead to stochastic extinction. Tumor eradication occurs due to stochastic events that drive tumor cells to extinction. We thus ask whether parameter variability or stochastic nature of the underlying process, or both, is responsible for the distribution of the time to cure and if they behave differently in the two models.

**Fig. 4:**
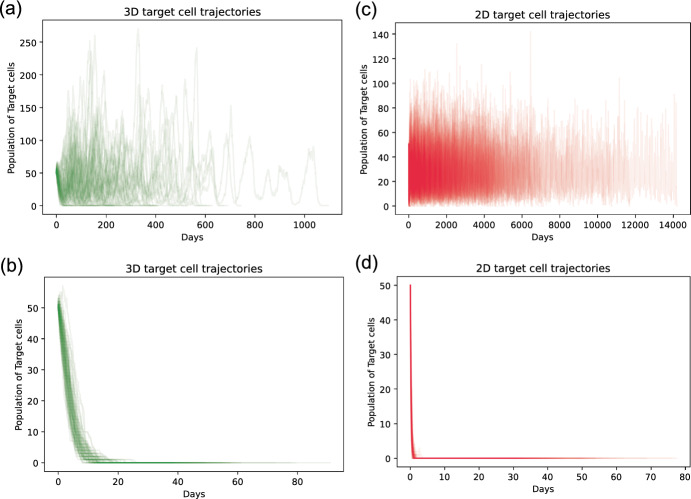
Stochastic simulation results - model with conjugate and model without conjugate tumor trajectories. Panels (**a**) and (**b**) show 100 tumor trajectories in the stochastic simulation results of the model with conjugate. Panels (**c**) and (**d**) describe those in the model without conjugate. The initial conditions are effector T cells $E= 30$, target tumor cells $T= 50$. For (**a**), we choose $\gamma =0.02$. In panel (**c**), we set the parameters $\beta _1=0.018$ and $\beta _2=0.002$. In panel (**b**), $\gamma$ value is increased to 0.2. For panel (**d**), we set the parameters $\beta _1=0.18$ and $\beta _2=0.02$ (the other parameters are $s=0.05$, $r=0.15$, $d=0.1$. In (**a**) and (**b**), specially $\alpha _1=0.4$ and $\alpha _2=0.05$)

Compared to the deterministic version of the model, the stochastic model can capture the dynamics for a smaller number of cells as every change in population is explicitly simulated. We use stochastic simulations to describe the time distribution of tumor eradication using the Gillespie algorithm. Figure 4 shows 100 simulations of tumour population trajectories in both models. Panel (c) displays long-time fluctuation until tumor extinction. We set trajectories to be slightly transparent, thus denser color represents more trajectories overlap. Most of the trajectories fluctuate and decrease to extinction before 6000 days. In this simulation, the two tumor and effector cell interaction rates $\beta _1$ and $\beta _2$ are relatively low; their sum is 0.02. This suggests that under this parameter setting, a tumor cell population in the system without conjugate can survive at a low amount for a long time before finally go extinct.

For the model with a conjugate compartment, in most cases the tumor cell population goes extinct more quickly, as Fig. 4a shows. However, the simulation results of both models show a similar trend when a larger value of tumor and effector cell binding rate $\gamma$ is chosen. In Fig. 4b, we choose the same value for all the parameters except increasing tumor and effector cell binding rate $\gamma$ tenfold to be 0.2. In panel (d), the sum of two rates $\beta _1$ and $\beta _2$ increases to 0.2. An increase of $\gamma$ prevents tumor trajectories from fluctuating; instead, nearly all trajectories decline sharply in the beginning, although trajectories from the model with conjugate reach extinction at a later time. We next investigate how the parameters affect the tumor extinction time distribution.

**Fig. 5:**
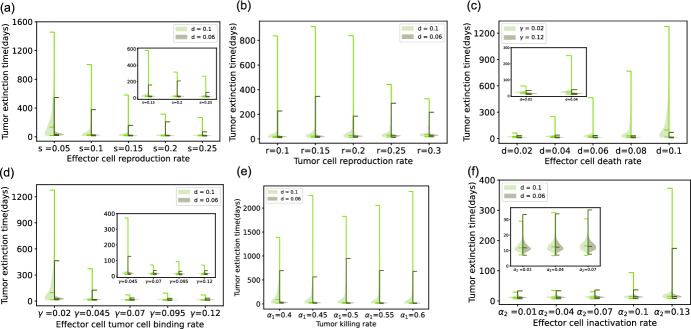
Stochastic simulation results - violin plots of tumor extinction time distribution for model with conjugate. The default parameters are $s=0.05$, $r=0.15$, $\gamma =0.02$, $\alpha _1=0.4$, and $\alpha _2=0.05$. In the left half part (green color), the effector cell death rate parameter is set to be $d = 0.1$ and for the right half (grey color), $d = 0.06$. The initial conditions are effector T cells $E= 50$, target tumor cells $T= 100$. Panel (**a**) displays tumor extinction time (in days) distribution violin plots for variation of the birth rate of effector cells *s*. In the inset, we zoom into the three distributions on the right. Note that here the tumor-killing rate is $\alpha _1=0.35$. Panel (**b**) displays tumor cell reproduction rate *r* variability and the extinction time distribution. Specially we set $s=0.1$ here. Panel (**c**) describes tumor extinction time distribution violin plots for parameter *d*, the natural death rate of effector cells. In the left half part (green color), effector cell and tumor cell binding rate parameter $\gamma$ equals 0.02 and in the right half part (grey color), $\gamma = 0.12$. The inset shows the left two distributions in more detail. In panel (**d**), effector cell and tumor cell binding rate parameter $\gamma$ is varied. The inset shows the right four distributions. In panel (**e**), the tumor-killing rate $\alpha _1$ varies. For panel (**f**), the effector cell exhaustion rate $\alpha _2$ is varied. The inset zooms into the three distributions on the left. We fix other parameters $\gamma =0.12$ and $\alpha _1=0.5$ (Color figure online)

We compare the tumor eradication time distribution and potential influence parameters in both model settings. We start from the situation where the tumor is in a stable coexistence steady state for both deterministic models. The maximum simulation time is set to be very large, 50000 days. This helps to capture the stochastic dynamics of tumor cells for even longer than a human lifetime. We either stop the simulation when the maximum time is reached or end if it fulfills a defined criterion, that is the occurrence of a stochastic mutation event, at a small rate $\mu = 10^{-6}$. We define the criterion as a mutation with a small rate of occurrence, as we assume the mutated tumor subclone has higher fitness and thus can lead to rapid proliferation. On the one hand, the mutated tumor changes the stochastic regime. On the other hand, it also drives the tumor’s successful escape. Six parameters are varied to see if the variability affects the tumor extinction time. Figure 5 show the violin plot of tumor cell eradication time distributions simulated from the model with conjugate. The maximum, minimum, and median are included. We vary the parameters’ value to see the trend and shape of the tumor eradication time distribution. By comparing the median in the violin plot, Fig. 5 shows that with the increase of effector cell reproduction rate, effector cell - tumor cell binding rate or tumor killing rate will lead to faster the tumor cell population going to extinction, which validates our intuition. The quicker the effector cell is generated, the quicker the tumor cell is bound and killed by the effector cell, and the shorter time the tumor cell population takes to go extinct.

In contrast, the larger the tumor cell reproduction rate, effector cell death rate, or effector cell inactivation rate, the slower the tumor cell goes extinct. This also matches our intuition. We also observe from Fig. 5 that the increase of effector cell inactivation rate only slightly benefits the tumor survival by looking at the median extinction time. However, the tumor reproduction rate does not help with the longer extinction time.

**Fig. 6:**
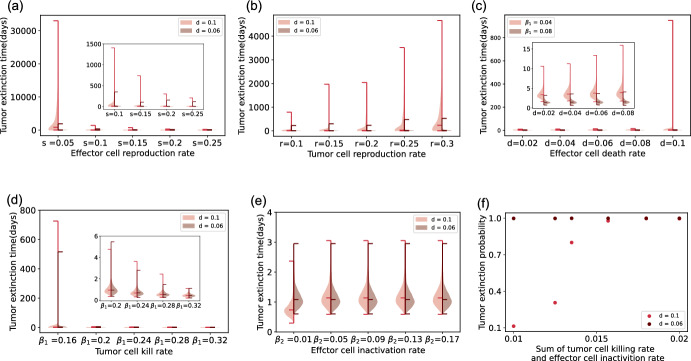
Stochastic simulation results - violin plots of tumor extinction time distribution for model without conjugate. The default parameters are $s=0.05$, $r=0.15$. In the left half part (pink), the effector cell death rate parameter *d* is set to be 0.1 and for the right half (brown), $d = 0.06$. The initial conditions are effector T cells $E= 50$, target tumor cells $T= 100$. Panel (**a**) displays tumor extinction time (in days) distribution violin plots for parameter *s*, the birth rate of effector cells. The inset zooms into the four distributions on the right. We set the other parameters $\beta _1=0.0175$ and $\beta _2=0.0025$. Panel (**b**) displays tumor cell reproduction rate *r* variability and extinction time distribution. Other parameters are fixed to $\beta _1=0.0178$, $\beta _2=0.0022$. Panel (**c**) describes tumor extinction time distribution violin plots for parameter *d*, the natural death rate of effector cells. In the left half part (pink rose color), effector cell and tumor cell binding rate parameter $\beta _1= 0.04$ and in the right half part (brown color), $\beta _1 = 0.08$. The inset shows the left four distributions in more detail. We fix $\beta _2=0.005$. In panel (**d**), tumor killing rate $\beta _1$ varies. In the inset, we zoom into the four distributions on the right. Specially $\beta _2=0.05$. In panel (**e**), the effector cell inactivation rate $\beta _2$ varies. We fix $\beta _1 = 0.16$. Panel (**f**) shows the tumor extinction probability. The sum of $\beta _1$ and $\beta _2$ vaires from 0.01 to 0.02 with the ratio of $\beta _1$ and $\beta _2$ fixed (Color figure online)

Some parameters influence extinction time distribution differently in the model without a conjugate compartment. The effector cell inactivation rate does not play an obvious role in tumor extinction time distribution. An increase of the two parameters *r* and *d* extends the tumor extinction time distribution, although the majority of tumor trajectories vanish at the beginning. Some parameters have the opposite influence, parameters *s* and $\beta _1$, representing effector cell reproduction rate and tumor cell killing rate; their increase leads to the shrink of the tumor cure time distribution. However, there are a number of tumor trajectories in the model without conjugate that take more time to be eliminated. Among the 1000 simulation runs, in Fig. 6a, for $s=0.05$ and $d=0.1$, four of the tumor trajectories fail to get extinction. Also, in Fig. 6b, for $r = 0.3$, $d = 0.1$, one run does not lead to tumor eradication. We investigate the role other parameters play in the tumor extinction probability. The sum of $\beta _1$ and $\beta _2$ plays a vital role in extinction probability. We set the ratio of $\beta _1$ and $\beta _2$ to be a constant $c = \frac{\beta _1}{\beta _2} = 0.125$. Then the sum of $\beta _1$ and $\beta _2$ is $(1+c)\beta _1$. The sum varies from 0.01 to 0.02; correspondingly, the value of $\beta _1$ varies from 0.0089 to 0.0178. The probability increases with the value of the sum. We summarise the extinction probability in Fig. 6f. We include an example tumor population trajectory plot in Appendix D Fig. 8a, in which the sum of $\beta _1$ and $\beta _2$ is small in the parameter setting. The extinction probability plot for parameter *d* can also be found in Appendix D.

For the model with conjugate, by studying the linear stability, we conclude that there is a chance for tumors to escape when the coexistence steady state is non-stable. However, in the stochastic regime, this leads to much more likely tumor extinction as the trajectory oscillation amplitude increases, which makes it easier to go extinct.

## Discussion

**Fig. 7:**
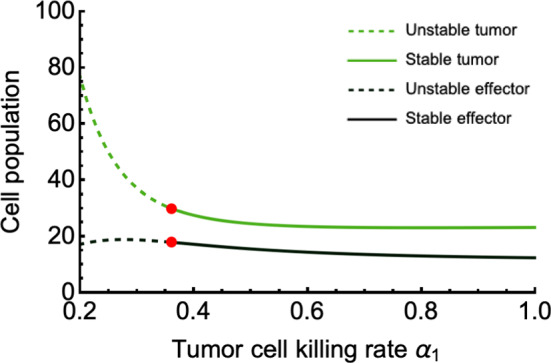
Bifurcation plot. The tumor cell population is green, and the effector cell population is dark green. Dashed lines are unstable fixed points and lines are stable fixed points. Red points indicate the bifurcation points (parameters $s=0.05$, $r=0.15$, $d=0.1$, $\gamma =0.01$, and $\alpha _2=0.01$) (Color figure online)

**Fig. 8:**
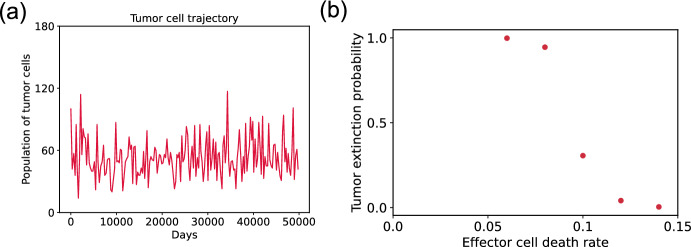
A tumor population trajectory that avoids extinction and extinction probability as a function of effector cell death rate. **a** an example realisation trajectory with $s=0.05$, $r=0.15$, $d=0.1$, $\beta _1=0.1$, and $\beta _2=0.0125$ for model without conjugate. For clarity of plotting, we display every 5000th time point. **b** Extinction probability plot for effector cell death rate *d*. Other parameter values are fixed the same as in (a) (Color figure online)

The formation of cellular conjugates can crucially impact cancer-immune dynamics on the cellular level. In this study, we investigated the importance of modeling the transition stage in tumor and effector cell interaction by comparing two models with and without explicitly considering the dynamics of a conjugate compartment. We characterized differences in the stability of the tumor and effector co-existence between these approaches. When conjugate dynamics are not explicitly considered but absorbed into the rates of killing and exhaustion, the tumor may reach stable coexistence with effector cells. However, this co-existence state can be altered if we explicitly consider the rate-adjusted equivalent model with conjugate compartment dynamics: the co-existence state can be unstable when the effector cell exhaustion rate is low, and the tumor-killing rate is sufficiently low. Therefore, tumor cells can escape from the immune surveillance by the effector cells. For sufficiently large effector exhaustion rates, the tumor-effector co-existence state is stable for any tumor-killing rate. In the stochastic regime, setting parameters that lead to a stable tumor-immune coexistence, tumor trajectories can go to extinction when conjugates are included. Figure 7 shows that the fixed point stability changes with the value of $\alpha _1$ when other parameters are fixed. We choose values from the range that the fixed points are stable for the stochastic simulation as we are interested in the extinction problem when the population is in stable equilibrium; on the other hand, from linear stability analysis, we know that in the model without conjugate, the co-existence (non-trivial) fixed point is stable, so it’s also necessary to keep the deterministic long term behavior consistent for comparing the extinction in the stochastic regime. For the model without conjugate, a small portion of tumor trajectories do not go extinct but oscillate at a low level throughout the simulated lifespan.

In the model with a conjugate compartment, it takes more time to reach the stable tumor-effector cell coexistence state. We conclude from Fig. 2d that the tumor and effector cell binding rate does not influence the damping rate of the oscillating ODE trajectories toward equilibrium, thus not influencing the time to reach the coexistence equilibrium state. However, the higher the tumor reproduction rate, the longer it takes to reach possible coexistence. Increasing any of the other four parameters (i.e., effector cell reproduction rate, effector cell death rate, tumor cell killing rate, and effector cell inactivation rate) reduces the time to converge to steady state. For the stochastic dynamics in the case of small population sizes, Fig. 5d illustrates that parameters such as tumor effector cell binding rate, which did not affect the stability of deterministic co-existence, led to faster tumor extinction as it increased. Compared to the model with conjugate, parameters in the approach without conjugate show a more pronounced influence on the extinction time distribution.

There are several limitations. Some parameter sets for specific cancers can be chosen to better understand the conjugate transition problem in different cancer types. The simplified assumption of 1 effector cell: 1 tumor cell can be loosened to investigate multiple effector cells binding one tumor cell and provide further insight.

Specially, we assumed that the reproduction term of the effector cell population positively correlates to the tumor cell density. This results from effector cells only recruiting by antigen-presenting cells’ simulation which conveys information about tumor cell existence. This reproduction term makes our model different from the classical predator–prey model.

Our work suggests that the dynamics of an immune cell-tumor cell conjugate compartment can be crucial for the understanding of possible tumor-control equilibria. The expected steady-state and stability may drastically change if this compartment is not explicitly considered. A possible co-existence state is unstable as it largely depends on the explicit parameter values in the model with a conjugate compartment. Thus, including a conjugate compartment, tumor populations can escape if the tumor-killing rate is sufficiently low. If tumor-immune conjugate dynamics are somewhat ignored because the transient conjugate compartment is assumed to be in a quasi-steady state (Kuznetsov et al. 1994), the rate of tumor escape is more challenging to predict. The pioneering work by Kuznetsov et al. (1994) focused on the quasi-steady-state model of the cytotoxic T lymphocyte response to the growth of tumor, assuming the conjugate dynamics is constant. The conjugate formation process before the effector T cell kills target tumor cells can be regarded as a handling time term for predator and prey models. The handling time here also defines how effector killing is reduced due to effector cell binding with a target tumor cell. Modeling the tumor-immune conjugation as a separate compartment allows for more flexibility regarding the possible outcomes of cancer-immune predation, such as tumor escape or tumor control.

Understanding the critical conjugation process in tumor-immune interactions will benefit the development of immunotherapies for predicting long-term outcomes such as tumor control or eradication. Our mathematical model provides a framework to describe the dynamics of small tumor populations emerging during early cancer evolution or after treatment, resulting in either extinction or progression. The work presented here takes a step toward demonstrating the importance of the conjugate compartment in defining tumor-effector interactions. Models of early-stage onco-immunological interactions taking transitionary compartments into account, may better capture the dynamics of tumor progression. For future modeling geared toward clinical data, considering the conjugate compartment may lead to improved insights in predicting tumor fates.

## Data Availability

The simulation code for this project is publicly available, https://github.com/paltrock/conjugates.

## Link between Stochastic and Deterministic Description

Here, we apply the Fokker-Planck approximation to study the same question model without the conjugate compartment between discrete and continuum regimes. Let $\mathbb {P}(i,j;t+d t)$ be the probability that there are *i* effector cells and *j* tumor cells at time $t+d t$. The master equation of the model without conjugate is:

6 $$\begin{aligned} \begin{aligned} \mathbb {P}(i,j;t+d t) =&\underbrace{ \mathbb {P}(i,j;t) (1-dt(i s+j r+i d+ij \beta _1+ij \beta _2) )}_\text {outflux}\\&+ \underbrace{dt((i-1) s \mathbb {P}(i-1,j;t)}_\text {influx: reproduce one effector cell}\\&+\underbrace{(j-1) r \mathbb {P}(i,j-1;t)}_\text {influx: reproduce one tumor cell} \\&+ \underbrace{(i+1) d \mathbb {P}(i+1,j;t)}_\text {influx:death of one effector cell} \\&+\underbrace{i(j+1) \beta _1 \mathbb {P}(i,j+1;t)}_\text {influx: kill one tumor cell} \\&+\underbrace{(i+1) j \beta _2 \mathbb {P}(i+1,j;t))}_\text {influx: inactivate one effector cell} \\&+O(dt^2) \end{aligned} \end{aligned}$$

We can write the probability of transition as: $s(i) = i s$, $r(j) = jr$, $d(i) = id$, $\beta _{1}(j) = \frac{i j \beta _1}{\Omega }$, $\beta _{2}(i) = \frac{i j \beta _2}{\Omega }$. Plugging in the transition probability and rearranging, we have:

7 $$\begin{aligned} \frac{\partial \mathbb {P}(i,j;t)}{\partial t}= -&\mathbb {P}(i,j;t)(s(i)+r(j)+d(i) +\beta _{1}(j)+\beta _{2}(i))\nonumber \\ +&s(i-1) \mathbb {P}(i-1,j;t)\nonumber \\ +&r(j-1) \mathbb {P}(i,j-1;t)\nonumber \\ +&d(i+1) \mathbb {P}(i+1,j;t) \nonumber \\ +&\beta _{1}(j+1) \mathbb {P}(i,j+1;t) \nonumber \\ +&\beta _{2}(i+1) \mathbb {P}(i+1,j;t) \end{aligned}$$

Rescaled variable with system size $\Omega$, scaled variable $x = \frac{i}{\Omega }$, $y = \frac{j}{\Omega }$.

The master equation becomes

8 $$\begin{aligned} \begin{aligned} \frac{\partial \mathbb {P}(x,y;t)}{\partial t}= -&\Omega (\mathbb {P}(x,y;t)(s(x)+r(y)+d(x)+\beta _{1}(y)+\beta _{2}(x))) \\ +&\Omega s\left( x-\frac{1}{\Omega }\right) \mathbb {P}(x-\frac{1}{\Omega },y;t)\\ +&\Omega r\left( y-\frac{1}{\Omega }\right) \mathbb {P}(x,y-\frac{1}{\Omega };t)\\ +&\Omega \beta _{1}\left( y+\frac{1}{\Omega }\right) \mathbb {P}(x,y+\frac{1}{\Omega };t)\\ +&\Omega \left( d\left( x+\frac{1}{\Omega }\right) +\beta _{2}\left( x+\frac{1}{\Omega }\right) \right) \mathbb {P}(x+\frac{1}{\Omega },y;t) \end{aligned} \end{aligned}$$

Expanding the master equation to $(\frac{1}{\Omega })^2$ (with $\frac{1}{\Omega }\ll 1$), we obtain the Fokker-Planck equation

9 $$\begin{aligned} \frac{\partial \mathbb {P}(x,y;t)}{\partial t} = - \left( f_x \frac{\partial \mathbb {P}}{\partial x} +f_y \frac{\partial \mathbb {P}}{\partial y}\right) +\frac{1}{2\Omega } \left( g_x^2 \frac{{\partial ^{2}}\mathbb {P}}{\partial x^{^2}} + g_y^2 \frac{{\partial ^{2}}\mathbb {P}}{\partial y^{^2}}\right) \end{aligned}$$

where $$g_x^2 = s(x)+d(x)+\beta _2(x)$$, $$g_y^2 = r(y) + \beta _1(y)$$, $$f_x = s(x)-d(x)-\beta _2(x)$$, $$f_y = r(y)-\beta _1(y)$$.

To connect with the ordinary differential equation, assuming $\Omega$ goes to infinity, we keep the first term, multiply both sides by *x*, and integrate over all possible values.

The left-hand side is:

$$\begin{aligned} \int \int x \frac{\partial \mathbb {P}}{\partial t} \,dx \,dy = \frac{d\langle x\rangle }{dt} \end{aligned}$$

The right-hand side is:

$$\begin{aligned} -\int \int x (f_x \frac{\partial \mathbb {P}}{\partial x} +f_y \frac{\partial \mathbb {P}}{\partial y}) \,dx \,dy=-\int x f_x\mathbb {P} \bigg |_{x =0}^{x =1} \,dy = \langle f_x\rangle \end{aligned}$$

as $\frac{\partial x}{\partial y} = 0$, the second term vanishes.

Similar computation for *y*, we obtain the equation:

10a $$\begin{aligned} \frac{d\langle x\rangle }{dt} =&\langle s x-d x-\beta _2 x y\rangle \end{aligned}$$

10b $$\begin{aligned} \frac{d\langle y\rangle }{dt} =&\langle r y- \beta _1 x y\rangle \end{aligned}$$

which is a re-scaled version of Eq. (4b).

Applying the same method, we can link the stochastic and deterministic model for the model with conjugate.

## Non-Dimensionalision and Characteristic Time

We non-dimensionalize Eq. (1c). assume there are four intrinsic units of measurement $E_0$, $T_0$, $C_0$ and $t_0$ with the same units as *E*, *T*, *C* and *t* respectively, such that these conditions hold:

$$\begin{aligned} \frac{t}{t_0}=\tau \end{aligned}$$

$$\begin{aligned} \frac{E}{E_0}=x \end{aligned}$$

$$\begin{aligned} \frac{T}{T_0}=y \end{aligned}$$

$$\begin{aligned} \frac{C}{C_0}=z \end{aligned}$$

Then the model can be rewritten as

11a $$\begin{aligned} \frac{\textrm{d}x}{\textrm{d}\tau }&=y-xy+z-x \end{aligned}$$

11b $$\begin{aligned} \frac{\textrm{d}y}{\textrm{d}\tau }&= p_1y-p_2xy+p_3z \end{aligned}$$

11c $$\begin{aligned} \frac{\textrm{d}z}{\textrm{d}\tau }&= p_4xy-p_5z \end{aligned}$$

where

12a $$\begin{aligned} p_1&= \frac{r}{d} \end{aligned}$$

12b $$\begin{aligned} p_2&= \frac{s}{d} \end{aligned}$$

12c $$\begin{aligned} p_3&= \frac{\alpha _2s}{\alpha _1d} \end{aligned}$$

12d $$\begin{aligned} p_4&= \frac{\alpha _1}{d} \end{aligned}$$

12e $$\begin{aligned} p_5&= \frac{\alpha _1+\alpha _2}{d} \end{aligned}$$

We find that *d* is the common denominator of all rescaled parameters, suggesting the characteristic time. Similarly, the non-dimensionalized equation for Eq. (4b) is

13a $$\begin{aligned} \frac{\textrm{d}x}{\textrm{d}\tau }&=y-xy-x \end{aligned}$$

13b $$\begin{aligned} \frac{\textrm{d}y}{\textrm{d}\tau }&= q_1y-q_2xy \end{aligned}$$

where

14a $$\begin{aligned} q_1 =&\frac{r}{d} \end{aligned}$$

14b $$\begin{aligned} q_2 =&\frac{\beta _1 s}{\beta _2 d} \end{aligned}$$

The common denominator of all rescaled parameters is also *d*.

## Stability of Steady States

### Model Without Conjugate

The model without conjugate, Eq. (3b) has two steady states. Here we prove that the non-trivial one is stable. The Jacobian is given by $$\begin{pmatrix} -d + \frac{d r \beta _2}{- s \beta _1 +r \beta _2} & s-\frac{r \beta _2}{\beta _1}\\ \frac{d r \beta _1}{-s \beta _1 + r \beta _2} & 0 \end{pmatrix}$$. The eigenvalues $\lambda _1$ and $\lambda _2$ are

15 $$\begin{aligned} \lambda _{1,2}=\frac{d \beta _1^2s \pm \sqrt{d}\beta _1\sqrt{-4\beta _2^2r^3+8\beta _1\beta _2r^2s+d\beta _1^2s^2-4\beta _1^2rs^2}}{2\beta _1(\beta _2r-\beta _1s)} \end{aligned}$$

We show that the real part of both eigenvalues is always negative. The fixed point only exists if $\beta _2r < \beta _1 s$, cf. 2. Therefore, the denominator is always negative. Thus, we have to show that the real part of the numerator of $\lambda _{1,2}$ is always greater or equal to 0.

There are two cases:

(i) If the argument of the square root, $-4\beta _2^2r^3+8\beta _1\beta _2r^2s+d\beta _1^2s^2-4\beta _1^2rs^2$, is negative, the real part of the two Eigenvalues is given by
  16 $$\begin{aligned} \lambda _{1,2}=\frac{d \beta _1^2s}{2\beta _1(\beta _2r-\beta _1s)}. \end{aligned}$$
  As the denominator is negative, and *d* and *s* are positive, the real part is negative.
(ii) If the argument of the square root, $-4\beta _2^2r^3+8\beta _1\beta _2r^2s+d\beta _1^2s^2-4\beta _1^2rs^2$, is positive, we need to show that
  17 $$\begin{aligned} d\beta _1^2s \ge \sqrt{d}\beta _1\sqrt{-4\beta _2^2r^3+8\beta _1\beta _2r^2s+d\beta _1^2s^2-4\beta _1^2rs^2} \end{aligned}$$
  We divide by $\sqrt{d}\beta _1$
  18 $$\begin{aligned} \sqrt{d}\beta _1s \ge \sqrt{-4\beta _2^2r^3+8\beta _1\beta _2r^2s+d\beta _1^2s^2-4\beta _1^2rs^2} \end{aligned}$$
  and square both sides
  19 $$\begin{aligned} d\beta _1^2s^2 \ge -4\beta _2^2r^3+8\beta _2\beta _1r^2s-4\beta _1^2s^2r + d\beta _1^2s^2. \end{aligned}$$
  We remove the term $d\beta _1^2s^2$ to arrive at
  20 $$\begin{aligned} 0 \ge r(-4\beta _2^2r^2+8\beta _2\beta _1rs-4\beta _1^2s^2) = -4r (\beta _2 r - \beta _1 s)^2, \end{aligned}$$
  which is always fulfilled. Thus, the numerator is always positive, and thus, the eigenvalues have a negative real part.

### Model with Conjugate

For the model with conjugate, we numerically solve the ordinary differential equations and plot the bifurcation diagram for tumor and effector cell populations and parameter tumor cell killing rate $\alpha _1$, shown in Fig. 7. We vary $\alpha _1$ from 0.2 to 1 to see the populations and the stability change.

## Extinction Probability

In Fig. 8a, we provide an example trajectory when the value of $\beta _1+\beta _2$ is small. We can observe the trajectory keeps fluctuating throughout time, thus avoiding extinction.

The extinction probability plot for *d* is displayed in Fig. 8b. The variation of the rest of the parameters does not affect the extinction probability significantly.
